# Draft Genome Sequence and Annotation of the Entomopathogenic Bacterium *Photorhabdus luminescens* LN2, Which Shows Nematicidal Activity against *Heterorhabditis bacteriophora* H06 Nematodes

**DOI:** 10.1128/genomeA.01268-14

**Published:** 2014-12-11

**Authors:** Xuehong Qiu, Zu-Bing Zhan, Xun Yan, Richou Han

**Affiliations:** Guangdong Entomological Institute, Guangdong Key Laboratory of Integrated Pest Management in Agriculture, Guangdong Public Laboratory of Wild Animal Conservation and Utilization, Guangzhou, China

## Abstract

We present here the 5.6-Mb genome sequence of *Photorhabdus luminescens* strain LN2, a Gram-negative bacterium that is a symbiont of *Heterorhabditis indica* LN2 and shows nematicidal activity against *Heterorhabditis bacteriophora* H06 nematodes.

## GENOME ANNOUNCEMENT

*Photorhabdus luminescens* is a species of Gram-negative bacteria that is pathogenic to insects and mutualistic with *Heterorhabditis* nematodes, providing us a model for the study of host-pathogen interactions and symbiosis (1–3). *P. luminescens* subsp. *akhurstii* LN2 is a symbiont of *Heterorhabditis indica* LN2 and shows nematicidal activity against *Heterorhabditis bacteriophora* H06 nematodes (4–6). Here, we present a draft genome sequence for *P. luminescens* strain LN2.

High-throughput Illumina sequencing technology was used to perform paired-end sequencing of a genomic DNA sample of *P. luminescens* LN2. After filtering the low-quality bases, we assembled the short reads into a genome sequence using SOAP*denovo* version 1.05 (http://soap.genomics.org.cn/soapdenovo.html). The final draft assembly contained 122 contigs and 85 scaffolds, with *N*_50_s of 186,732 bp and 297,028 bp, respectively. These contigs and scaffolds were obtained with 5,586,746 and 5,596,724 bp, respectively. Both have a G+C content of 42.8%. GLIMMER (7) was used for gene prediction, with default settings, and the open reading frames (ORFs) at the boundaries of the scaffolds and those covering two or more scaffolds were excluded. We discovered 5,306 ORFs, of which the average length is 879 bp. The total length of the gene regions is 4,659,057 bp, accounting for 83.2% of the genome. The G+C content of the gene regions is 44.3%. Two rRNA genes, 78 tRNA genes, and 13 small RNA (sRNA) genes were predicted by RNAmmer, tRNAscan-SE1.21, and Rfam, respectively (8–10).

Rapid Annotations using Subsystems Technology (RAST) (11) results showed that LN2 contains the genes for all the essential pathways for carbohydrate, DNA, RNA, and protein metabolism. There are also genes involved in the metabolism of fatty acids, lipids, and isoprenoids, of which some lipases, one class of secreted proteins, may be involved with insect pathogens. Genes involved in iron acquisition and metabolism are also present in the genome annotation of LN2 and may contribute to the adaptation of low-iron conditions in insects.

Since the genome sequence of *P. luminescens* TT01 was obtained (12), more and more genomes of strains of *Photorhabdus* and *Xenorhabdus* have been sequenced (13–18). Together with the sequences of these genomes, the genome sequence of LN2 may provide us with new insights into the mechanisms underlying pathogenicity and mutualism. Because the mutualism between nematodes and bacteria is safe, effective and commercial bioinsecticides for many economically important pests (5), studying them will help us develop new and better bioinsecticides.

### Nucleotide sequence accession numbers.

This whole-genome shotgun project has been deposited at DDBJ/EMBL/GenBank under the accession no. JQOC00000000. The version described in this paper is version JQOC01000000.
